# Characterizing Early Maternal Style in a Population of Guide Dogs

**DOI:** 10.3389/fpsyg.2017.00175

**Published:** 2017-02-10

**Authors:** Emily E. Bray, Mary D. Sammel, Dorothy L. Cheney, James A. Serpell, Robert M. Seyfarth

**Affiliations:** ^1^Department of Psychology, University of PennsylvaniaPhiladelphia, PA, USA; ^2^Department of Biostatistics and Epidemiology, Perelman School of Medicine, University of PennsylvaniaPhiladelphia, PA, USA; ^3^Department of Biology, University of PennsylvaniaPhiladelphia, PA, USA; ^4^Department of Clinical Studies, School of Veterinary Medicine, University of PennsylvaniaPhiladelphia, PA, USA

**Keywords:** maternal style, canine, guide dogs, nursing, licking/grooming, behavior

## Abstract

In both humans and non-humans, differences in maternal style during the first few weeks of life can be reliably characterized, and these differences affect offspring's temperament and cognition in later life. Drawing on the breeding population of dogs at The Seeing Eye, a guide dog school in Morristown, New Jersey, we conducted videotaped focal follows on 21 mothers and their litters (*n* = 138 puppies) over the first 3 weeks of the puppies' lives in an effort to characterize maternal style. We found that a mother's attitude and actions toward her offspring varied naturally between individuals, and that these variations could be summarized by a single principal component, which we described as *Maternal behavior*. This component was stable across weeks, associated with breed, litter size, and parity, but not redundant with these attributes. Furthermore, this component was significantly associated with an independent experimental measure of maternal behavior, and with maternal stress as measured by salivary cortisol. In summary, *Maternal behavior* captured a significant proportion of the variation in maternal style; was stable over time; and had both discriminant and predictive validity.

## Introduction

In rodents and primates, early separation from the mother has been shown to play a negative role in offspring's later expression of emotions, as measured by task performance and glucocorticoid receptor density (e.g., Aisa et al., 2008); later behavior, as measured by fear conditioning (e.g., Kosten et al., 2006); and later cognition, as measured by performance in inhibitory control tasks (e.g., Pryce et al., 2004). However, simply having a mother present is not enough; her behavior toward her offspring also matters. Individual differences in maternal styles are both distinguishable and important for later outcomes. For example, in Old World monkeys, mothers naturally fall along separate scales of infant rejection (tolerance of contact, carrying, and nursing) and protection (levels of grooming, proximity, and tendency to limit offspring exploration; e.g., Fairbanks, 1996; Parker and Maestripieri, 2011). In rodents, mothers vary in the amount of licking-grooming (LG) and arched-back nursing (ABN) that they display toward their offspring (e.g., Liu et al., 2000; Meaney, 2001; Fish et al., 2004).

Importantly, these variations in maternal style have real biological and cognitive consequences for the offspring. Rhesus macaque offspring who face repeated rejection by their mothers have lower levels of serotonin later in life (Parker and Maestripieri, 2011). Rodent pups raised by high LG-ABN mothers show better spatial memory (Liu et al., 2000) and exhibit muted stress responses when compared to offspring raised by low LG-ABN mothers (Francis et al., 1999). Researchers have implicated neural mechanisms through which high levels of early maternal care, and specifically tactile stimulation, might lead to superior spatial cognition and altered stress responses. Prominently featured in most theories is the hippocampus, which is widely acknowledged to play a role in spatial memory (e.g., Clayton and Krebs, 1994). High levels of early stimulation by the mother are thought to lead to increased NMDA receptors, which in turn facilitate the release of brain-derived neurotrophic factor and hippocampal synaptogenesis (e.g., Meaney, 2001; Fish et al., 2004). Liu et al. (2000) found significantly more NMDA receptors in the rat offspring of high LG-ABN mothers as early as 8 days and continuing into adulthood. Thus, it would appear that, above and beyond the presence of a mother figure during early development, the content and quality of interaction that the mother provides is extremely important for later outcomes.

Despite their close association with humans, comparatively little is known about the effects of maternal style on the cognitive and emotional development of domestic dogs (Czerwinski et al., 2016b; Serpell et al., 2016). In dogs, mothers provide care for at least the first 5 weeks postpartum and puppies reach sexual maturity in roughly 6–12 months (Morey, 1994). This relatively abbreviated life history makes dogs an ideal candidate in which to study the effects of maternal care. And yet surprisingly little progress has been made in characterizing early maternal style or documenting its effects on later offspring behavior, temperament, and outcomes. Questionnaire studies reveal that dog breeders attach little to no importance to maternal care when considering breeding stock (Leroy et al., 2007; Czerwinski et al., 2016a). However, as results from the broader animal literature indicate that maternal care is extremely important, understanding its effects in dogs specifically is crucial. Given that many working dog organizations breed their own animals in highly controlled and supervised environments, determining the types of maternal behaviors that lead to favorable outcomes and then encouraging and selecting for those maternal styles could be a highly effective strategy for increasing program graduation rates.

The few studies that have examined maternal care in dogs are consistent with the results from other species. For example, when puppies weaned at 4–6 weeks were compared with those weaned at 8–12 weeks, puppies weaned later were less prone to behavioral problems at older ages (Fält and Wilsson, 1979; Pierantoni and Verga, 2007; Pierantoni et al., 2011), reinforcing the view that maternal separation can have negative effects on behavior. Similarly, using a questionnaire, Tiira and Lohi (2015) had owners rate mothers on a scale of one (low levels of maternal interest) to seven (high levels of interest), and found that lower levels of maternal care were linked to fear and anxiety in offspring as adults.

In two recent studies, investigators monitored differences in mothers' natural interactions with their puppies over the first 3 weeks of life. Foyer et al. (2016) monitored mother-pup interactions of German Shepherd dogs bred to be military working dogs, using five variables associated with the time that mothers spent in contact and interacting with their puppies. These variables loaded onto one principal component, which was associated with pup temperament at up to 18 months of age. In a similar study of beagles, Guardini et al. (2016) used four variables to assess mother-pup behavior during a daily 15-min period for the first 3 weeks of life. Again, these variables reduced into a single principal component reflecting amount of maternal care, which was significantly correlated with measures of puppy temperament at 2 months.

Our goal in the current study was to categorize the variations in maternal behavior during the first 3 weeks after birth and to use independent concurrent measures of behavior and hormones to validate our observations. We hypothesized that if there are consistent behavioral differences among mothers' interactions with their puppies, these differences might be associated both with maternal behavior in other contexts and with maternal cortisol levels. This method of validating a behavioral profile with behaviors and physiological factors that were not considered when building the profile has been used previously in studies of primates (Seyfarth et al., 2012).

Subjects were mothers and puppies bred to be guide dogs for the blind and visually impaired. We chose this population for several reasons. First, they provided us with a large sample of subjects that were housed and reared under controlled conditions, allowing for systematic observations. Cooperation of The Seeing Eye® personnel gave us access to background data on breed, age, and parity, allowed uninterrupted access to the same dogs across a variety of ages, and permitted us to conduct targeted experiments and standardized hormonal measurements. Finally, by around 16 months of age, the puppies in our study entered training as Seeing Eye® dogs, a process that led to either success or failure in the program. The problem-solving skills demanded of guide dogs are extremely complex, with exacting temperamental as well as cognitive requirements (Johnston, 1990). Guide dogs need to be sufficiently motivated to learn and tackle tasks, but also calm enough to rest quietly for hours at a time while their handlers are at work. Furthermore, they must filter the sensory information they encounter, giving full attention to relevant material (e.g., the flow of traffic) while ignoring the rest (e.g., passersby attempting to pet or play). They also need to differentiate among subtle commands (e.g., “wait” [short period of time] vs. “stay” [long period of time]); appropriately navigate environmental stimuli (e.g., escalators, revolving doors, curbs); resist temptation (e.g., dropped food on a restaurant floor); and respond to unanticipated events (e.g., an open manhole in the sidewalk, a closure along the usual route). The long-term goal of our research, therefore, was to examine possible associations between maternal behavior, measures of temperament and cognition in young adult dogs, and ultimate success in the Seeing Eye® program. The present paper constitutes a first step, by characterizing variation in maternal style. In upcoming papers (Bray et al., unpublished data), we examine the relationship between early maternal style and offspring performance on tests of temperament and cognition around 16 months, as well as offspring's outcome in the Seeing Eye® program.

In Part 1 of the study, we monitored maternal interactions in 21 litters that included three different breeds. We found that variation in seven behavioral measures could be summarized by one principal component, called *Maternal behavior*. Because breed, litter size, birth season, parity, and litter sex ratio have been identified in previous studies as potentially affecting both maternal behavior (Guardini et al., 2015; Foyer et al., 2016) and/or later puppy behavior (e.g., Borchelt, 1983; Wilsson and Sundgren, 1998; van der Waaij et al., 2008; Foyer et al., 2013, 2016), we then tested for associations between these variables and maternal style.

In Part 2, we validated our measure of maternal style by testing its association with experimental measures of maternal preference for puppies over a human visitor.

Finally, in Part 3, we searched for associations between maternal style and maternal stress levels before and after a brief separation from their puppies. By describing observed maternal style and ensuring it was related to other measures within the same timeframe, we aim to generate data that will provide the starting point for explicit predictions in subsequent work.

## General methods

Mothers and puppies were housed at the breeding facility at The Seeing Eye, Inc. (Morristown, NJ, USA), a philanthropic organization that breeds, raises, and trains guide dogs for the blind and visually impaired. Mothers and puppies were the property of The Seeing Eye, Inc., which granted informed consent to all aspects of the study. All testing procedures adhered to regulations set forth and approved by the University of Pennsylvania Institutional Animal Care and Use Committee (Protocol #805210).

The mothers in the study had all spent the first year and a half of their life with volunteer puppy-raisers where they received extensive socialization. During this time, the dogs were provided with basic obedience training, taken on field trips, and exposed to as many environments, people, and objects as possible. Dogs chosen to be breeders then completed 2 months of guide dog training and were handpicked to enter the breeding program based on their health, behavior, and genetic diversity.

At the breeding station, adult dogs were walked daily by volunteers and assigned to a staff member who continued to train them in basic obedience and agility. They were housed socially with one to two kennelmates in two adjoining 9′ by 10′ indoor pens, with access to two adjoining 9′ by 12′ outside pens. Pens were arranged in a circle so that dogs had visual access to all other pens and all humans that entered the room. About 1 week prior to their whelping date, mothers were transferred from the breeding wing to the whelping wing of the facility. Housing conditions here were similar to those in the breeding station, except that mothers were now housed singly. The inside pen was equipped with a round hard plastic kiddie pool (6′ diameter × 1′ tall) lined with towels, which served as the whelping pool. Post-birth, puppies remained in the whelping pool for the first 3 weeks. While training was discontinued for the 6 weeks that mothers spent in the whelping wing, mothers still received daily exercise and attention from volunteers and staff. Mothers remained in the whelping wing until their puppies were weaned at 5 weeks postpartum, at which point they returned to the breeding wing of the facility. Mothers received food 3 times a day and water was always available. The lights were switched on around 6:30 and turned off at 23:00^1^.

Over the study period (February–August 2014), 23 mothers whelped at the breeding station. Two of these mothers were excluded from the study due to atypical circumstances: one for having only one puppy and one for being separated from her litter over long periods prior to weaning. Thus, our final sample consisted of 21 mothers (nine German Shepherds, eight Labrador Retrievers, and four Golden Retriever; see Table 1) and their litters (*n* = 138 puppies).

**Table 1:** **Mother and litter demographics**.

**Mother**	**Breed**	**Age (months)**	**Parity**	**Litter size**	**Litter breed**
Della	Labrador Retriever	49	4	6	Labrador-Golden Cross
Lizzie	Golden Retriever	22	1	9	Golden
Dagmar	German Shepherd	46	3	8	German Shepherd
Dori	Golden Retriever	29	2	5	Labrador-Golden Cross
Lolly	German Shepherd	37	3	2	German Shepherd
Dotty	Golden Retriever	29	2	2	Labrador-Golden Cross
Onyx	Labrador Retriever	33	2	8	Labrador Retriever
Maude	Labrador Retriever	23	1	9	Labrador Retriever
Ayesha	Labrador Retriever^*^	32	2	10	Labrador Retriever
Foxy	German Shepherd	31	2	7	German Shepherd
Toffee	Labrador Retriever	57	5	6	Labrador Retriever
Carey	Labrador Retriever	31	2	8	Labrador Retriever
Aura	German Shepherd	42	3	7	German Shepherd
Naomi	Labrador Retriever^*^	40	3	8	Labrador Retriever
Omega	Golden Retriever	40	3	8	Golden Retriever
Lea	German Shepherd	49	4	6	German Shepherd
Leah	German Shepherd	37	3	5	German Shepherd
Paris	German Shepherd	26	2	4	German Shepherd
Elise	German Shepherd	23	1	9	German Shepherd
Xyris	Labrador Retriever	40	3	7	Labrador Retriever
Lisa	German Shepherd	38	3	4	German Shepherd
Mean and S.D.		35.9 ± 9.2	2.6 ± 1.0	6.6 ± 2.2	

* *These dogs are Labrador-Golden Crosses × 2, meaning their mothers were 50–50% Labrador-Golden Crosses and their sires were 100% Labrador Retrievers, making them 75% Labrador Retriever. Thus, in all analyses, these dogs were classified as Labrador Retrievers*.

## Part 1: observations of maternal style

### Methods

During the post-whelping period when mothers were in the same pen with their litters, our goal was to characterize maternal style by measuring variation in behavior across mothers. We aimed to create a maternal style profile of each mother through intensive video observations, allowing for later comparison of these results to experimental and endocrine measures collected in parallel.

#### Subjects and procedure

We monitored each of the 21 mothers' behavior for 3 weeks (days 1–21) after birth. Three days per week, we videotaped two 10-min sessions in the morning (between 9:00 and 12:00) and two 10-min sessions in the afternoon (between 15:00 and 18:00). We used Sony video cameras (HDR-PJ230, HDR-CX405) mounted on tripods outside of individual pens. Video footage was stored on hard drives and later analyzed using Datavyu (DatavyuTeam, 2014). We only recorded videos when the mother and all of her puppies were present in the pen, all humans were absent from the pen, and the mother was not eating. We coded a total of 6890 min of footage. Because access to monitoring was sometimes temporarily suspended due to cleaning, kennel staff, volunteer presence, or unforeseen circumstances (i.e., mothers whelping), the total video footage per litter over the 3 weeks ranged from 150 to 360 min (mean = 328 min, see Table 2 for observation time of individual mothers).

**Table 2:** **Mother and litter participation**.

**Mother**	**Observation time: Week 1 (minutes)**	**Observation time: Week 2 (minutes)**	**Observation time: Week 3 (minutes)**	**Reaction to human testing (week observed)**	**Maternal cortisol (week collected)**
Della	110	0	40	3	NA
Lizzie	110	0	120	3	NA
Dagmar	0	120	90	2, 3	2
Dori	120	100	120	1, 2, 3	1, 2
Lolly	100	120	120	1, 2, 3	1, 2
Dotty	100	120	120	1, 2, 3	2
Onyx	120	120	80	1, 2, 3	1, 2
Maude	120	120	120	1, 2, 3	1, 2
Ayesha	120	120	120	1, 2, 3	1, 2
Foxy	120	120	120	1, 2, 3	1, 2
Toffee	120	120	80	1, 2, 3	1, 2
Carey	120	120	120	1, 2, 3	1, 2
Aura	120	120	120	1, 2, 3	1, 2
Naomi	120	120	80	1, 2, 3	1, 2
Omega	120	120	120	1, 2, 3	1, 2
Lea	120	120	120	1, 2, 3	1, 2
Leah	120	120	120	1, 2, 3	1, 2
Paris	120	120	120	1, 2, 3	1, 2
Elise	120	120	120	2, 3	1, 2
Xyris	120	120	120	1, 2	1
Lisa	120	120	120	1, 2, 3	1, 2

#### Scoring

Most coded variables were chosen based on their inclusion in past studies examining mothering in dogs (Guardini et al., 2015; Foyer et al., 2016). Moreover, because the rodent literature has successfully tracked different nursing postures and discovered associations with later pup behavior (e.g., Myers et al., 1989b; Liu et al., 2000; Champagne et al., 2003) and because our own observations revealed behavioral differences in nursing postures among the mothers, we diverged from past dog studies that coded nursing as a single category and instead coded distinct types of nursing. The following seven variables were coded from video:

*Time in pool:* Mother's whole body in whelping pool.
*Vertical nursing per pup:* Mother nursing (at least one puppy suckling) while standing or sitting in the whelping pool, divided by the number of puppies in her litter.
*Lateral nursing per pup:* Mother nursing (at least one puppy suckling) while lying on her side or back, so that part or all of her nipples were exposed, divided by the number of puppies in her litter.
*Ventral nursing per pup:* Mother nursing (at least one puppy suckling) while lying on her stomach, so that her nipples were not easily exposed to the puppies, divided by the number of puppies in her litter.
*Contact per pup:* Mother lying in close proximity to one or more of her puppies so that the puppies' bodies and/or faces were touching the mother, divided by the number of puppies in her litter.
*Licking/grooming per pup:* Mother licking, grooming, and/or sniffing her puppies, divided by the number of puppies in her litter.
*Orienting out:* Mother orienting/looking outside of her pen to the main area of the pavilion. This behavior was only coded when the mother was in the whelping pool with her puppies.

For each of these seven variables, we calculated the average duration in seconds of the behavior during each week's observation sessions. Thus, each of the 21 mothers ended up with one score on all seven variables from each of the 3 weeks (Table 2). Exceptions were Della (missing week 2), Lizzie (missing week 2), and Dagmar (missing week 1).

Coding from video (*n* = 689 10-min sessions) was completed by four different observers, with the majority (55%) coded by EB. Three other observers, each of whom had participated in all aspects of the study, coded 15% of the videos. Inter-rater reliability was determined by randomly selecting 15 10-min sessions for all observers to code. A high degree of reliability was found for all variables [ICC (2,4) = 0.96–1.00 with a 95% confidence interval from 0.89 to 1.00]. We were unable to distinguish individual puppies from video, so our observation unit was the litter (as in Foyer et al., 2016).

#### Statistical analysis

All statistical analyses were carried out in R version 3.3.0 (R Development Core Team, 2016). We first examined the raw scores on the seven behavioral variables and tested for rank-order stability over time using Kendall's rank correlation coefficients.

To summarize variation in maternal behavior, we applied principal components analysis (PCA) with orthogonal (varimax) rotation to the seven behavioral variables. We first verified that there was an adequately compact pattern of correlations, indicated by Kaiser-Meyer-Olkin (KMO) measures of sampling adequacy >0.5, and that our variables were sufficiently correlated, indicated by a significant Bartlett's test (*p* < 0.05). We determined the number of factors to retain by using parallel analysis (Horn, 1965), fit using the R package “paran” (Dinno, 2012), as well as the Comparison Data technique (Ruscio and Roche, 2012). Each mother received a score on each principal component on weeks 1, 2, and 3. We tested for rank-order stability in principal component scores over time using Pearson correlation coefficients.

Finally, because breed, litter size, birth season, parity, and litter sex ratio have been identified in previous studies as potentially affecting mother and puppy behavior, we included each of these parameters as covariates in the regression model described below. Mother's age was not included as it was redundant with parity (*r* = 0.97, *p* < 0.001). To test for associations between these covariates and the single factor, *Maternal behavior*, that best explained our data, we used a Generalized Estimating Equations version of a general linear regression model (GEE-GLM) to estimate adjusted associations of breed, litter size, birth season, parity, and litter sex ratio with *Maternal behavior*. We used a Gaussian error distribution with litter as the unit of analysis. Variance estimates for the statistical tests on the regression coefficients were adjusted for clustering due to litter effects using generalized estimating equations (Liang and Zeger, 1993). All models were fit using the R package “geepack” (Halekoh et al., 2006). To compare models, we used the R package “anova” in the “stats” package.

### Results

#### Stability of *maternal behavior* over time

As in previous studies (e.g., Foyer et al., 2016), the average of all seven variables of maternal care decreased over time. With few exceptions, the rank orders of mothers on the different behaviors were significantly correlated (i.e., stable) from weeks 1 to 2 and 2 to 3 (Table 3).

**Table 3:** **Consistency of maternal behavior across weeks**.

**Behavior**	***n***	**Time frame**	**Kendall's tau**
Time in pool	18	Weeks 1–2	0.341^*^
	19	Weeks 2–3	0.590^***^
Contact per pup	18	Weeks 1–2	0.245
	19	Weeks 2–3	0.419^*^
Licking/grooming per pup	18	Weeks 1–2	0.305·
	19	Weeks 2–3	0.371^*^
Lateral nursing per pup	18	Weeks 1–2	0.322·
	19	Weeks 2–3	0.430^*^
Ventral nursing per pup	18	Weeks 1–2	0.302
	19	Weeks 2–3	0.033
Vertical nursing per pup	18	Weeks 1–2	0.462^*^
	19	Weeks 2–3	0.624^**^
Orienting out	18	Weeks 1–2	0.541^**^
	19	Weeks 2–3	0.274

*** *p < 0.001*,

** *p < 0.01*,

* *p < 0.05, ·p < 0.10*.

#### Principal components analysis

A KMO test was conducted on the seven behavioral variables listed above. The sampling adequacy for the analysis was KMO = 0.69. All KMO values for individual variables were >0.54, which is above the acceptable limit of 0.5 (Field et al., 2012). Bartlett's test, χ^2^(21) = 221, *p* < 0.001, indicated that correlations between items were sufficiently large for PCA. Furthermore, all items were correlated to at least the level of 0.49 with at least one other item, suggesting reasonable factorability. Parallel analysis using 5000 iterations recommended retaining one principal component, as did the Comparison Data technique. The scree plot showed inflexions that were consistent with retaining a one-component solution. The total variance explained by this factor was 54% (Table 4).

**Table 4:** **Components and loadings of the PCA over the observations of maternal care**.

**Observation variable**	***Maternal behavior***
Time in pool	0.72
Contact per pup	0.90
Licking/grooming per pup	0.72
Lateral nursing per pup	0.84
Vertical nursing per pup	0.54
Ventral nursing per pup	0.63
Orienting out	0.74
Eigenvalue	3.78
Proportion of variance explained	0.54

Loadings of the behavioral variables onto the principal component (*Maternal behavior*) suggested that mothers who scored high on this component were often present and interactive with their puppies. They spent a considerable amount of time in the pool, were in frequent contact with their puppies, and showed high levels of oral behavior toward their puppies, including anogenital licking, grooming of the face and body, and sniffing. They often nursed them from a lateral position that provided puppies with the easiest access to their milk, but also engaged in nursing from a ventral or vertical position that made it more labor intensive for puppies to nurse effectively. They also were often attentive toward the main pavilion rather than toward their puppies (orienting out), despite being in the pool with them.

#### Stability of *maternal behavior* over time

The rank orders of scores on *Maternal behavior* were significantly correlated (i.e., stable) from weeks 1 to 2 (*r* = 0.82, *p* < 0.001) and 2 to 3 (*r* = 0.89, *p* < 0.001).

#### Potential covariates

We conducted a GEE-GLM with breed (a categorical variable), litter size (2–10), birth season (a binary variable consisting of spring versus winter), parity (1–5), and litter sex ratio (percent male) as the predictors and *Maternal behavior* as the dependent measure (Table 5). *Maternal behavior* was not related to birth season or litter sex ratio. However, Labrador Retriever mothers demonstrated significantly higher *Maternal behavior* scores than German Shepherds (β = 0.56, *SE* = 0.19, *p* = 0.003). Additionally, mothers with smaller litters had significantly higher *Maternal behavior* scores than mothers with larger litters (β = −0.32, *SE* = 0.07, *p* < 0.001), and mothers who had whelped fewer litters had significantly higher *Maternal behavior* scores than more experienced mothers (β = −0.16, *SE* = 0.07, *p* = 0.02).

**Table 5:** **Results of a GEE-GLM in which the dependent variable was *Maternal behavior***.

**Predictor variables**	**Estimate**	***SE***	***p*****-value**
Intercept	2.43	0.52	<0.001^***^
Golden Score	−0.11	0.31	0.716
Labrador Score	0.56	0.19	0.003^**^
Birth season	0.35	0.28	0.215
Litter sex ratio	−0.53	0.40	0.184
Litter size	−0.32	0.07	<0.001^***^
Parity	−0.16	0.07	0.018^*^

Predictor variables were Golden score, Golden Retriever compared to German Shepherd; Labrador score, Labrador Retriever compared to German Shepherd; birth season, spring or winter; litter sex ratio, percent male; litter size, 2–10; and parity, 1–5). Mother ID was entered as a random effect. N = 17 mothers (week 1) and 18 mothers (week 2). Statistical tests of significance use GEE.

*** *p < 0.001*,

** *p < 0.01*,

* *p < 0.05*.

“Contact per pup” was the behavior that loaded most strongly onto *Maternal behavior*, and it was also associated with (a) litter size: mothers with smaller litters had more contact per pup than mothers with larger litters (β = −0.31, *SE* = 0.06, *p* < 0.001), (b) breed: Labrador Retriever mothers had more contact per pup with their litters than German Shepherd mothers (β = 0.56, *SE* = 0.15, *p* < 0.001), and (c) parity: mothers who had whelped fewer litters had more contact with their pups than more experienced mothers (β = −0.22, *SE* = 0.07, *p* < 0.001). The loading of “contact per pup” was not just an artifact of these covariates, however, because a model that used *Maternal behavior* score along with litter size, parity, and breed to predict the frequency with which a mother lay in contact per pup was significantly better than a model that used only litter size, parity, and breed [ANOVA model comparison, χ^2^(1) = 30.2, *p* < 0.001]. In other words, mothers who scored high on *Maternal behavior* were in contact with their puppies more frequently than would have been expected based only on their litter size, breed, and parity.

We therefore concluded that the principal component was not redundant with breed, litter size, birth season, parity, or sex ratio. However, because some associations were found with these covariates, we included them as potential confounding variables in subsequent models.

In sum, we tracked seven maternal care behaviors of 21 mothers toward their litters over the first 3 weeks post-whelping. The mothers' interactions were best explained by one principal component of maternal style, which was stable over time. *Maternal behavior* was not explained by birth season or sex ratio of the litter. While *Maternal behavior* was associated with breed, litter size, and parity, this component still provided additional information above and beyond that provided by those demographic variables alone.

## Part 2: experimental test of maternal style

### Methods

Assuming that *Maternal behavior* captured individual variation in maternal style that was consistent over time, we hypothesized that it should also be associated with other measures of maternal behavior that were collected concurrently but were not used to generate the principal component. To that end, we conducted a 5 min preference test that sought to quantify a mother's attentiveness to her puppies in the face of a competing social interest: a familiar human in the whelping pen. The design of this experiment was meant to evoke the mothers' typical interactions with kennel staff, since staff members entered each pen multiple times per day. The goal was, first, to place the mothers in a situation where they would have to choose between their puppies and an inviting distraction, and then to determine if the mothers' choices could be predicted based on their mothering style as measured by their scores on the PC.

#### Subjects and procedure

The same mothers that were observed above participated in this experiment, conducted once a week during their first (*n* = 17 litters), second (*n* = 19 litters), and third (*n* = 21 litters) weeks postpartum (see Table 2).

All experiments took place between 8:45 and 18:30. When the mother was in the pool nursing at least one puppy and no other humans were in the pavilion, the experimenter set up the camera outside of her pen, started the camera, and left the area. One minute later, the experimenter returned. First, she greeted the dog verbally by name once, outside of the gate. Immediately afterwards, she opened the gate and greeted the dog again from inside the pen. Then for 1 min she stood in the pen, facing toward the pool from ~2 feet away, in a relaxed posture. If the mother left the pool and interacted with the experimenter during this time, the experimenter responded by petting and talking to the mother. For the next minute, the experimenter sat on the ground in the same spot. Again, the experimenter interacted with the mother if the mother approached her. Finally, for the last minute, the experimenter remained sitting where she was but hid her face and actively ignored the mother. After a total of 3 min had elapsed, the experimenter exited the pen and the session ended. The experimenter was always one of three females of similar age, with the first author (EB) playing the role of experimenter in 95% of all sessions.

#### Scoring

The following three variables were coded from video:

*Initial approach:* Time elapsed (in seconds) between the experimenter's first greeting and when all four of the mother's paws were outside of the whelping pool. In order to approach the human, the mother had to stop nursing and leave her puppies. A low time corresponded with the mother being least interested in her puppies while in the presence of the familiar human, while a high time corresponded to the mother being most invested in remaining with her puppies. If a mother never left her puppies over the course of the experiment, she received the maximum score of 180 s.
*Contact with human:* Duration (in seconds) that the mother was outside of the pool and sniffing or touching the experimenter with any part of her body, ranging from 0 to 168 s.
*Time with puppies:* Percent of the 3 min session that the mother remained in the pool with her puppies (all four legs in pool) while the experimenter was in the pen, ranging from 0 to 100%.

Each of the 21 mothers received scores on all three variables from each of the 3 weeks, with the following exceptions: Della (missing weeks 1 and 2), Lizzie (missing weeks 1 and 2), Dagmar (missing week 1), Elise (missing week 1), and Xyris (missing week 3; Table 2).

All coding was completed by an observer who had not participated in data collection. To assess reliability of the video-coded measures, EB then coded 20% of randomly selected trials. Ratings on initial approach and time with puppies were correlated at r_τ_ = 1 (*p* < 0.001), and ratings on contact with human were correlated at r_τ_ = 0.94 (*p* < 0.001).

#### Statistical analysis

All statistical analyses were carried out in in R (version 3.3.0, R Foundation for Statistical Computing, R Development Core Team, 2016). The three outcome variables were standardized using a *z*-score because they used different scales. Mothers received a score on each outcome variable during weeks 1 (*n* = 17), 2 (*n* = 19), and 3 (*n* = 21). We used Pearson correlation coefficients to determine how associated the outcome measures were, and found them all highly correlated (*r* > 0.90). We therefore chose one and tested for rank-order stability on that measure over time using a Pearson correlation coefficient. We then used a GEE-GLM that included the data for each week and used litter as the unit of observation to test whether *Maternal behavior* was associated with a mother's relative interest in her puppies, as measured by our one outcome variable. We included as predictors an interaction, *Maternal behavior* by week, as well as breed, litter size, birth season, parity, and litter sex ratio. We then used a backward-selection strategy and retained all confounders that influenced the association of interest by at least 10%.

### Results

#### Outcome variables

All three outcome variables were highly correlated with one another. “Initial approach” was correlated with “Contact with human”: *r* = −0.91, *p* < 0.001 and with “Time with puppies”: *r* = 0.95, *p* < 0.001. “Contact with human” was correlated with “Time with puppies”: *r* = −0.94, *p* < 0.001. In other words, mothers who were slow to initially approach the experimenter also spent short amounts of time in contact with the experimenter and a considerable amount of the 3 min experimental session in the pool with their puppies. Because “Time with puppies” was the variable most highly correlated with the other two, we used that measure as the dependent variable in subsequent analyses.

The rank order scores of mothers on “Time with puppies” from weeks 1 to 2 were positively associated (*r* = 0.28), but not significant (*p* = 0.30). The same pattern held true from weeks 2 to 3 (*r* = 0.16, *p* = 0.50).

#### Maternal interest in puppies during the experiment

We were interested in evaluating if our PC, *Maternal behavior*, was associated with this experimental measure of a mother's relative interest in her puppies, as measured by her “Time with puppies” score.

We used a GEE-GLM with “Time with puppies” as the dependent variable, *Maternal behavior* as a predictor variable, and mother ID as a random effect. We also included an interaction, *Maternal behavior* by week, to test whether the relationship between maternal style and a mother's reaction to a human visitor changed as the puppies got older. Finally, we included breed, litter size, birth season, parity, and litter sex ratio as covariates. We were able to remove litter sex ratio and litter size from the final model, as they were determined to be non-significant and non-confounding (Table 6).

**Table 6:** **Results of a GEE-GLM in which the dependent variable was “Time with puppies”**.

**Predictor variables**	**Estimate**	***SE***	***p*****-value**
Intercept	1.89	0.34	<0.001^***^
Week 2	−0.94	0.24	<0.001^***^
Week 3	−1.16	0.29	<0.001^***^
Golden Score	0.02	0.31	0.937
Labrador Score	−0.33	0.22	0.136
Parity	−0.34	0.11	0.002^**^
Birth season	−0.58	0.27	0.013^*^
Interaction	0.58	0.22	0.007^**^
*Maternal behavior* × Week 1	0.05	0.13	0.710
*Maternal behavior* × Week 2	0.43	0.22	0.053^*^
*Maternal behavior* × Week 3	−0.15	0.16	0.334

*Predictor variables were Maternal behavior; week (1, 2, and 3); Golden score, Golden Retriever compared to German Shepherd; Labrador score, Labrador Retriever compared to German Shepherd; parity, 1–5; and birth season, spring or winter. Mother ID was entered as a random effect. N = 17 mothers (week 1), 19 mothers (week 2), and 21 mothers (week 3). Statistical tests of significance use GEE*.

*** *p < 0.001*,

** *p < 0.01*,

* *p < 0.05*.

The interaction was significant (β = 0.58, *SE* = 0.22, *p* < 0.01). Specifically, during week 2, mothers' scores on *Maternal Behavior* predicted how much time they spent with their puppies vs. the human (high scores predicted high preference for puppies; Week 2 β = 0.43, *SE* = 0.22, *p* = 0.05). There was no significant association during weeks 1 or 3 (Week 1 β = 0.05, *SE* = 0.13, *p* = 0.71; Week 3 β = −0.15, *SE* = 0.16, *p* = 0.33).

In sum, we conducted a weekly experiment to measure mothers' attention to their puppies in the face of a competing social interest. Mothers' responses were best characterized by her “Time with puppies” score. Mothers who scored high on *Maternal behavior*, i.e., those that regularly spent the most time in the whelping pool physically contacting, nursing, and grooming their puppies in their daily lives, also spent the most time with their puppies during week 2 of the experiment.

## Part 3: salivary cortisol reaction to a stressor

### Methods

To test the hypothesis that our PC, *Maternal behavior*, was associated with a measure of maternal stress, we compared this variable to salivary cortisol levels taken from the mother during the first 2 weeks after birth. Cortisol was collected before each mother experienced a brief 5 min separation from half of her litter, as well as 20 and 40 min after the separation. This scenario mimicked a potentially stressful, albeit typical event, as staff members removed neonatal puppies individually or in small groups to weigh them each day. This separation and the accompanying saliva collections were conducted on 2 nights each week.

We opted to measure cortisol from saliva because it is a noninvasive alternative to drawing blood (Beerda et al., 1996). Saliva collection closely adhered to previously published methods (Dreschel and Granger, 2009). While one or two experimenters held the dog in place, the main experimenter (EB) wore latex gloves and held a Salimetrics® Children's Swab under the dog's tongue and around the dog's cheek pouches, avoiding the gums, for 2–6 min. The swab was gently moved and repositioned throughout sampling, and then placed into a labeled storage tube. Past research indicates that handling of a dog during saliva collections lasting up to 5 min does not influence the cortisol concentration (Kobelt et al., 2003; Coppola et al., 2006), and in our own data mean cortisol levels were not correlated with duration of collection (*r* = 0.002, *p* = 0.97).

Post-collection, all samples were either refrigerated (at 4°C) and then centrifuged, or placed directly into the Triac™ centrifuge for 15 min at 1500x g rotation. After centrifugation and no more than 95 min after collection, samples were stored in a freezer (at −20°C) until 1–6 months later, when they were sent for analysis to Arizona State University's Institute for Interdisciplinary Salivary Bioscience Research.

#### Subjects and procedure

We obtained samples from mothers during their first (*n* = 17 mothers) and second (*n* = 18 mothers) weeks post-whelping (Table 2). Thus, all mothers who participated in the maternal separation experiment gave saliva on 4 separate days, with the exception of Dagmar (missing week 1), Lolly (missing day 2), Dotty (missing week 1), Onyx (missing days 2 and 4), Maude (missing day 2), Elise (missing day 4), and Xyris (missing day 2 and week 2).

Ten minutes prior to saliva collection, water was temporarily removed from the pen to avoid dilution of saliva. EB first collected baseline saliva between 20:00 and 21:00, ~2–3 h after the mother's evening meal, when she was in the pen with all of her litter. Between 21:00 and 23:00, an experimenter returned and removed half of her litter by placing them in a towel-lined tub and carrying them out of sight to the examination room located within the pavilion. Five minutes later, the experimenter returned the puppies to the mother. Then, 20 and 40 min after the initial removal, EB returned to collect post-stressor saliva from the mother. After the third saliva collection, water was returned to the mother and she received a Milk-Bone® biscuit.

Samples (*N* = 212, drawn from 19 dogs) were mailed on dry ice, then thawed and assayed for salivary cortisol using an enzyme immunoassay kit at the Institute for Interdisciplinary Salivary Bioscience Research. With the exception of one sample assayed as a singlet, all samples were assayed in duplicate using 50 μL of saliva, and the average of these two measures were used in subsequent analyses. The lower limit of detection was 0.007 μg/dl, and the average coefficient of variation for the assay was 3.0%. Analysis was repeated for five samples that had a coefficient of variation >15%.

#### Scoring

Prior to analysis, we applied a natural log transformation to all cortisol measurements. We also created a peak cortisol score for each mother on every collection day. Past studies in dogs and humans suggest that there is inter-individual variation in time to reach peak salivary cortisol levels (e.g., Beerda et al., 1996; Lopez-Duran et al., 2009). Thus, the peak cortisol score was calculated by subtracting each mother's baseline pre-stressor cortisol measure from her highest post-stressor cortisol measure, taken either 20 or 40 min after the removal of her puppies.

#### Statistical analysis

All statistical analyses were carried out in R (version 3.3.0, R Foundation for Statistical Computing, R Development Core Team, 2016). We first determined the best way to group the cortisol results by using a linear mixed model (LMM) to examine trends over time. We then tested if the mothers' baseline stress levels were associated with *Maternal behavior* by using a GEE-GLM. Finally, we tested if removing the puppies elicited a significant stress response across mothers by conducting a paired-samples *t*-test to compare the mean of the baseline scores against the mean of the peak scores. Upon finding a significant difference, we used a GEE-GLM to test whether the mothers' stress responses were associated with *Maternal behavior*. As before, we adjusted for confounding variables by using a backward-selection strategy and retained all confounders that influenced the association of interest by at least 10%.

### Results

#### Stability of cortisol over time

To determine if a mother's baseline cortisol levels differed by collection day, we used a LMM with baseline cortisol as the dependent variable, day (1 through 4) as the predictor variable, and dog ID as a random effect. Once again, day was not significant, indicating that mothers' baseline scores were similar across days.

We therefore created a Week 1 baseline cortisol score by taking the average scores across days 1 and 2 and a Week 2 baseline cortisol score by taking the average scores across days 3 and 4. For the four cases in week 1 and two cases in week 2 where a mother had only a single baseline score, we used that score alone.

To determine if a mother's peak cortisol response differed by collection day, we used peak cortisol score as the dependent variable, day (1 through 4) as the predictor variable, and dog ID as a random effect. Day was again not significant, indicating that mothers' cortisol responses were similar across all days.

This similarity in peak cortisol scores within weeks justified the creation of Week 1 and Week 2 average peak cortisol scores. For the three cases in week 1 and one case in week 2 where a mother had only a single peak cortisol score, we used that score alone.

#### Baseline maternal stress levels

We first asked if a mother's baseline cortisol scores were associated with *Maternal behavior*. We used a GEE-GLM with baseline cortisol score as the dependent variable, *Maternal behavior* as a predictor variable, and mother ID as a random effect. We also included an interaction of *Maternal behavior* by week, and breed, litter size, birth season, parity, and litter sex ratio as covariates. The interaction was nonsignificant, so it was dropped from the model. We were also able to exclude week, parity and litter sex ratio from the final model (Table 7).

**Table 7:** **Results of a GEE-GLM in which the dependent variable was the baseline cortisol score**.

**Predictor variables**	**Estimate**	***SE***	*p***-value**
Intercept	−2.21	0.28	<0.001^***^
*Maternal behavior*	0.16	0.09	0.061·
Golden Score	0.20	0.30	0.498
Labrador Score	0.07	0.17	0.664
Litter Size	0.05	0.05	0.269
Birth season	0.16	0.17	0.329

*Predictor variables were Licking/grooming; Golden score, Golden Retriever compared to German Shepherd; Labrador score, Labrador Retriever compared to German Shepherd; litter size, 2–10; and birth season, spring or winter. Mother ID was entered as a random effect. N = 17 mothers (week 1) and 18 mothers (week 2). Statistical tests of significance use GEE*.

*** *p < 0.001, ·p < 0.10*.

We found a marginally significant main effect (β = 0.16, *SE* = 0.09, *p* = 0.06): mothers who spent more time engaging in maternal behaviors also had higher levels of baseline cortisol over weeks 1 and 2.

#### Maternal response to separation

To verify that removing half of the puppies was indeed a stressful event for mothers, we compared the means of mothers' baseline cortisol scores to the means of mothers' peak cortisol scores. There was a significant difference between baseline scores (*M* = −1.73, *SD* = 0.41) and peak cortisol scores (*M* = 0.28, *SD* = 0.39); *t*_(34)_ = 21.28, *p* < 0.001.

Next, we investigated if a mother's anxiety when temporarily separated from half of her litter, as measured by her peak cortisol score collected during this event, was associated with the maternal style PC.

We used a GEE-GLM with the peak cortisol score as the dependent variable, *Maternal behavior* as a predictor variable, and mother ID as a random effect. We also included an interaction, *Maternal behavior* by week, to explore whether the relationship between a mother's investment in her puppies and her endocrine response differed from week 1 to 2. We also included breed, litter size, birth season, parity, and litter sex ratio as covariates, but were able to exclude parity and litter size from our final model (Table 8). We found a significant interaction (β = −0.23, *SE* = 0.09, *p* = 0.013): mothers who spent more time engaging in maternal behaviors tended toward higher levels of peak cortisol over week 1 (β = 0.20, *SE* = 0.11, *p* = 0.06), but this association disappeared by week two (β = −0.03, *SE* = 0.07, *p* = 0.68).

**Table 8:** **Results of a GEE-GLM in which the dependent variable was the peak cortisol score**.

**Predictor variables**	**Estimate**	**SE**	***p*****-value**
Intercept	0.00	0.17	0.991
Week	−0.02	0.14	0.900
Golden Score	0.08	0.15	0.605
Labrador Score	−0.14	0.10	0.146
Birth season	0.19	0.10	0.046^*^
Litter sex ratio	0.49	0.24	0.041^*^
Interaction			0.013^*^
*Maternal behavior* × Week 1	0.20	0.11	0.063·
*Maternal behavior* × Week 2	−0.03	0.07	0.675

*Predictor variables were Licking/grooming, week (1 or 2); Golden score, Golden Retriever compared to German Shepherd; Labrador score, Labrador Retriever compared to German Shepherd; litter sex ratio, percent male; and birth season, spring or winter. Mother ID was entered as a random effect. N = 17 mothers (week 1) and 18 mothers (week 2). Statistical tests of significance use GEE*.

* *p < 0.05, ·p < 0.10*.

To test the hypothesis that differences in maternal peak cortisol response were not simply a function of differences in maternal baseline cortisol levels, we ran the same full model as above but also included baseline cortisol as a predictor. Baseline was not significant in this model, and all other findings were consistent with our previous model, indicating that peak cortisol response was not just an artifact of baseline cortisol levels.

In sum, we measured maternal stress levels before and after a slightly stressful event. Pre-stressor, baseline salivary cortisol of the mothers over weeks 1 and 2 was positively associated with *Maternal behavior*. We also found that maternal peak salivary cortisol, or the maximum amount that a mother's cortisol levels increased from baseline levels following temporary puppy removal, was marginally associated with *Maternal behavior*. Specifically, mothers who ranked high on contact, proximity, nursing, grooming, and orienting out in both weeks showed the largest peaks in salivary cortisol after being briefly separated from half of their litter.

## General discussion

Consistent with results from previous studies (Foyer et al., 2016; Guardini et al., 2016), our observational data on seven measures of maternal style were best summarized by one principal component, *Maternal behavior*. Mothers who scored high on this component were present (often in close proximity and contact with their puppies), interactive (engaged in high levels of licking, grooming, and lateral, ventral, and vertical nursing), and vigilant (displayed high levels of orienting out into the main pavilion). This PC was also associated with concurrent behavioral and stress-related measures of mothering, results that have not been documented previously.

We validated our approach in several ways. First, mothers were consistent in their rank order on the PCs over time despite group-level decreases in maternal care as the weeks progressed. This stability gave us confidence that the PC captured meaningful and enduring individual differences. Results are also encouraging because they mirror data from the human and non-human literature, in which studies have found stable maternal differences over time (e.g., Berman, 1990; van IJzendoorn et al., 2000; Pittet et al., 2014).

Second, scores on PCs were not simply redundant with other measures, such as breed, litter size, birth season, parity, or litter sex ratio. If measures of maternal style had not provided any additional information beyond that provided by the demographic characteristics of mothers and their litters, then the extra time spent observing the mothers and building the profiles would not have been justified.

That being said, we did find some associations between our demographic covariates and *Maternal behavior*. In line with the results of Foyer et al. (2016), we found a significant effect of litter size, wherein females with smaller litters displayed higher scores on *Maternal behavior*. This is not surprising, since it is physically difficult for a mother of 10 to interact with each individual puppy at the same rate as a mother of two. Furthermore, tending to larger litters is likely more demanding and exhausting, potentially leading the mother to spend less time with them. Priestnall (1972) proposed that these aversive factors might be the reason that mice mothers of large litters spend less time in the nest than mothers of small litters, after finding that the difference could not be entirely explained by unequal nutritional requirements.

We also found an effect of parity: the more experienced the mother was, the less maternal behavior she displayed. In the only other study to examine the effect of parity on mother-pup interaction, Guardini et al. (2015) found that primiparous mothers increased their quality of care over time, surpassing multiparous mothers only in week 3, while multiparous mothers stayed relatively constant in their amount of care.

We were particularly interested in examining the effect of breed on *Maternal behavior*, as past studies have limited their sample to a single breed (Foyer et al., 2016; Guardini et al., 2016). We did find a breed difference among levels of *Maternal behavior*, since Labrador Retriever mothers spent more time engaging in maternal behavior than did German Shepherd mothers. No differences were found between Golden Retrievers and the other breeds, a result that might have been due in part to the small sample of this breed in our study.

In a third analysis that validated our approach, we found that the *Maternal behavior* PC had predictive validity, both in an experiment designed to test temporary maternal preference for puppies versus a familiar human, and in measures of maternal stress. Specifically, in an experiment designed to capture maternal preferences, we found a significant, time-dependent relationship between *Maternal behavior* and a mother's willingness or lack thereof to stay with her puppies in the presence of a familiar human, a behavioral measure that was not incorporated into the PC. Mothers who scored high on *Maternal behavior* in week 2 showed a high motivation to remain with their puppies, despite the presence of a human in the pen. This association was not present in the first and third weeks. The lack of an association during week 1 might be due to a ceiling effect: only 4 of the 17 mothers left the pool at all during the first week.

One limitation of this experimental measure is that not all dogs are equally social. Thus, if a female is not very socially motivated, she might stay in the pool with her pups even if her “maternal urge” is not very strong. However, in this population, we do not believe this concern affected our results. We found that, as expected, females who exhibited high levels of *Maternal behavior* were indeed more likely to stay with their puppies rather than visiting the human. One possible reason is that all of the dogs at The Seeing Eye have been bred over multiple generations for a friendly, human-oriented demeanor, whereas much less intense directional selection has been applied to mothering style.

In a fourth and final experimental validation of the predictive value of our components, we found two relationships between the PC and an independent physiological measure. First, we found a marginally significant positive association between *Maternal behavior* and higher baseline cortisol levels over weeks 1 and 2. This result suggests a link between high levels of maternal care and anxiety, although it is not yet known if this association might be detrimental to future litter outcomes. Additionally, we found an association between *Maternal behavior* and a measure of maternal stress response measure. Mothers who scored high on *Maternal behavior* in week 1 showed a larger stress response after half of their litter was temporarily removed, suggesting that puppy removal was particularly stressful for them. These findings are particularly interesting as past research has mainly focused on how maternal style influences later stress responses of the pups themselves (e.g., Francis et al., 1999; Myers et al., 1989a), and has investigated to a lesser extent how the stress of the mother might influence her maternal style (e.g., Champagne and Meaney, 2006). Moreover, these topics have primarily been explored in rodents.

Notably, we found the strongest associations between *Maternal behavior* and experimental measures of behavior and physiology during the first and second week postpartum. This result is consistent with findings from the rodent literature, where the first week after birth seems to have a particularly important effect on subsequent offspring behavior. In rats, for example, week 1 is the only week during which dams differ in their amount of licking and grooming (Champagne et al., 2003).

Although this study suggests that mothering style in dogs is consistent across weeks, we do not yet know if mothering style is consistent across litters. Data from rodents (Champagne et al., 2003), primates (Fairbanks, 1996), and sheep (Dwyer and Lawrence, 2000) suggest that maternal style is consistent across parturitions. It also remains to be determined how much the surrounding environment affects mothering profiles, as well as if and how maternal style can be manipulated to promote better offspring behavior, temperament, and cognition. In birds (Pittet et al., 2014) and macaques (Maestripieri, 1993), maternal style appears to be correlated with behavioral measures of the mother thought to be associated with temperament. We do not yet know if temperament in dogs can similarly be used to estimate maternal investment.

One surprising finding was the fact that all three nursing styles loaded onto the same component, although lateral nursing loaded the highest (Table 4). We had initially coded them separately because they were a striking feature of our preliminary observations, and because studies of rodents have distinguished three types of nursing (e.g., Myers et al., 1989b; Champagne et al., 2003). Importantly, only arched-back nursing has been consistently associated with later positive outcomes. Rat pups raised by high licking-grooming (LG) and arched-back nursing (ABN) mothers exhibited decreased startle responses and more exploratory behaviors in novel environments (e.g., Liu et al., 1997; Caldji et al., 1998). They also differed in measures of cognition; high LG-ABN pups exhibited superior object recognition (Meaney, 2001; Fish et al., 2004) and were faster and more efficient at problem-solving in a task for spatial memory (Liu et al., 2000) when compared with low LG-ABN pups. One hypothesis argues that these positive outcomes derive from the high levels of tactile simulation during the frequent nipple switching that occurs specifically during arched-back nursing (Liu et al., 2000). Arched-back nursing is also highly correlated with licking-grooming in rodents (Meaney, 2001), making it difficult to disentangle the separate effects; in fact, arched-back nursing is rarely measured by itself. Among the dogs in our study, however, vertical nursing and licking/grooming were only weakly correlated at 0.34. Dogs, therefore, may provide an opportunity to examine the separate roles of arched-back nursing and licking-grooming in affecting later behavior, as well as the effects of the three different nursing styles (Bray et al., unpublished data).

Several important questions remain. Now that we have established a reliable characterization of maternal behavior, we plan to test specific hypotheses about its effects on the later behavior of puppies, as well as investigating how enduring those effects may be. Recent studies of maternal care in dogs have found associations between higher levels of early care and increased exploratory tendencies and lower stress responses at 8 weeks (Guardini et al., 2016), as well as increased social engagement, physical engagement, and aggression at 15–18 months (Foyer et al., 2016). Long-term effects of maternal care have also been documented in primates; rhesus monkeys with rejecting mothers were more anxious at 2 years old (Maestripieri et al., 2006), and were also more independent than peers raised by protective mothers (Bardi and Huffman, 2002). Even in our own species, protective (DeVore and Ginsburg, 2005) and harmful (Repetti et al., 2002; Hoeve et al., 2009) effects on later adolescent behavior can be traced, at least in part, back to parenting style. In addition to these behavioral effects, early parental care has been linked to morphological changes in the brain. For example, one study in humans found that having warm, available parents at age four was correlated with hippocampal volume at age 14 (Rao et al., 2010). In addition to puppy temperament outcomes, future analyses will explore maternal effects on later cognition and problem-solving skills, as well as the ultimate success rates of puppies in The Seeing Eye® program.

## Author contributions

EB, RS, DC, and JS designed the observational and experimental procedures. EB conducted the observations, experiments, and coded the majority of the videos. EB, RS, and MS performed the statistical analyses. EB was the primary author, with assistance from RS and DC and comments from JS and MS.

## Funding

This work was supported in part by the University of Pennsylvania Department of Psychology's Norman Anderson Graduate Student Fund, a University of Pennsylvania University Research Foundation award, the Class of 1971 Robert J. Holtz Endowed Fund for Undergraduate Research, and a National Science Foundation Graduate Research Fellowship to EB (DGE-1321851). Any opinions, findings, and conclusions or recommendations expressed in this material are solely the responsibility of the authors and do not necessarily reflect the views of the National Science Foundation.

### Conflict of interest statement

The authors declare that the research was conducted in the absence of any commercial or financial relationships that could be construed as a potential conflict of interest. The reviewer FR and the handling Editor declared their shared affiliation, and the handling Editor states that the process nevertheless met the standards of a fair and objective review.

## Footnotes

^1^For additional information, see this website: http://bit.ly/2h9N3na and these informational videos: https://www.youtube.com/watch?v=7tJeqIJbTjg and https://www.youtube.com/watch?v=iI9MW6Hw5Fk.
